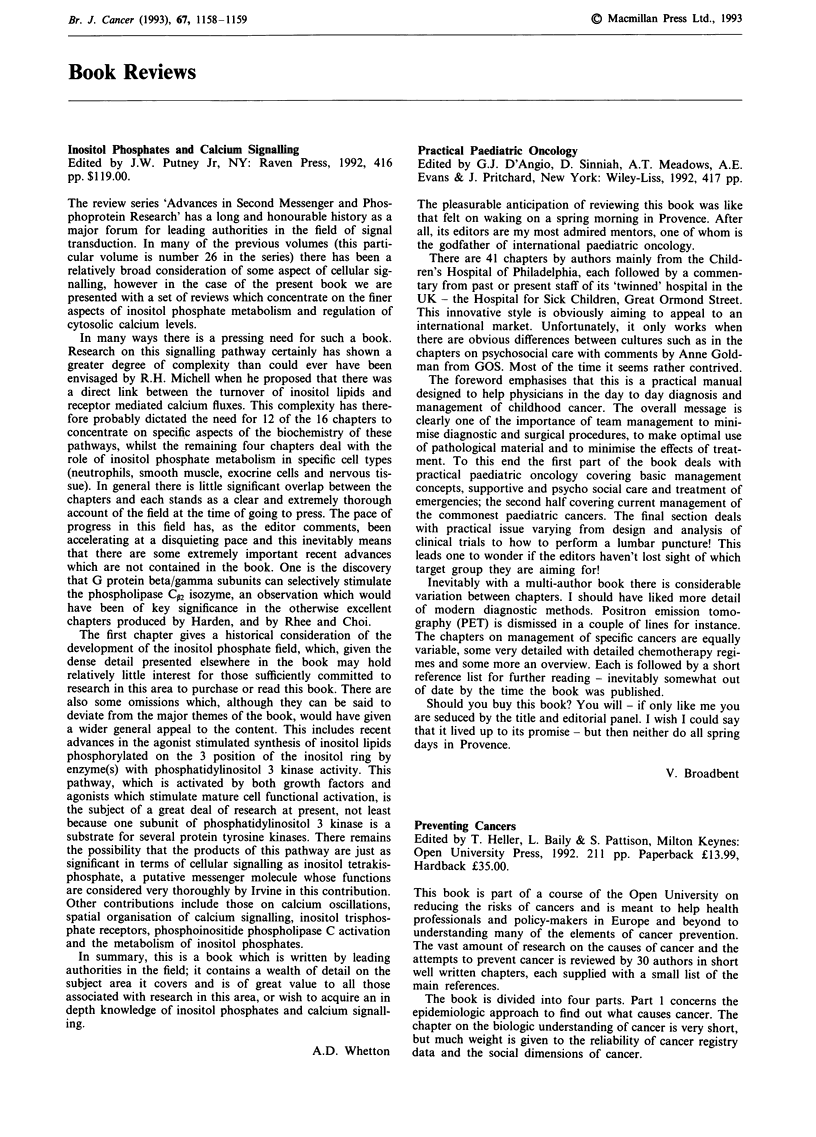# Inositol Phosphates and Calcium Signalling

**Published:** 1993-05

**Authors:** A.D. Whetton


### Book Reviews

**Inositol Phosphates and Calcium Signalling**
Edited by J.W. Putney Jr, NY: Raven Press, 1992, 416 pp. $119.00.

The review series 'Advances in Second Messenger and Phosphoprotein Research' has a long and honourable history as a major forum for leading authorities in the field of signal transduction. In many of the previous volumes (this particular volume is number 26 in the series) there has been a relatively broad consideration of some aspect of cellular signalling, however in the case of the present book we are presented with a set of reviews which concentrate on the finer aspects of inositol phosphate metabolism and regulation of cytosolic calcium levels.

In many ways there is a pressing need for such a book. Research on this signalling pathway certainly has shown a greater degree of complexity than could ever have been envisaged by R.H. Michell when he proposed that there was a direct link between the turnover of inositol lipids and receptor mediated calcium fluxes. This complexity has therefore probably dictated the need for 12 of the 16 chapters to concentrate on specific aspects of the biochemistry of these pathways, whilst the remaining four chapters deal with the role of inositol phosphate metabolism in specific cell types (neutrophils, smooth muscle, exocrine cells and nervous tissue). In general there is little significant overlap between the chapters and each stands as a clear and extremely thorough account of the field at the time of going to press. The pace of progress in this field has, as the editor comments, been accelerating at a disquieting pace and this inevitably means that there are some extremely important recent advances which are not contained in the book. One is the discovery that G protein beta/gamma subunits can selectively stimulate the phospholipase $C_{\beta 2}$ isozyme, an observation which would have been of key significance in the otherwise excellent chapters produced by Harden, and by Rhee and Choi.

The first chapter gives a historical consideration of the development of the inositol phosphate field, which, given the dense detail presented elsewhere in the book may hold relatively little interest for those sufficiently committed to research in this area to purchase or read this book. There are also some omissions which, although they can be said to deviate from the major themes of the book, would have given a wider general appeal to the content. This includes recent advances in the agonist stimulated synthesis of inositol lipids phosphorylated on the 3 position of the inositol ring by enzyme(s) with phosphatidylinositol 3 kinase activity. This pathway, which is activated by both growth factors and agonists which stimulate mature cell functional activation, is the subject of a great deal of research at present, not least because one subunit of phosphatidylinositol 3 kinase is a substrate for several protein tyrosine kinases. There remains the possibility that the products of this pathway are just as significant in terms of cellular signalling as inositol tetrakisphosphate, a putative messenger molecule whose functions are considered very thoroughly by Irvine in this contribution. Other contributions include those on calcium oscillations, spatial organisation of calcium signalling, inositol trisphosphate receptors, phosphoinositide phospholipase C activation and the metabolism of inositol phosphates.

In summary, this is a book which is written by leading authorities in the field; it contains a wealth of detail on the subject area it covers and is of great value to all those associated with research in this area, or wish to acquire an in depth knowledge of inositol phosphates and calcium signalling.

A.D. Whetton